# How to Measure the Mental Health of Teachers? Psychometric Properties of the GHQ-12 in a Large Sample of German Teachers

**DOI:** 10.3390/ijerph19159708

**Published:** 2022-08-06

**Authors:** Sarah Susanne Lütke Lanfer, Ruth Pfeifer, Claas Lahmann, Alexander Wünsch

**Affiliations:** 1 Department for Psychosomatic Medicine and Psychotherapy, Medical Center–University of Freiburg, Faculty of Medicine, University of Freiburg, 79104 Freiburg, Germany; 2 Department of Medical Oncology, Inselspital, Bern University Hospital, University of Bern, 3010 Bern, Switzerland

**Keywords:** teacher, mental health, prevention, factor structure, burnout, culture

## Abstract

To improve the health status of teachers, there is a need for good and reliable instruments to continuously assess their mental health. The current study proposed the GHQ-12 questionnaire as an appropriate instrument for measuring the mental health of teachers. The GHQ-12 is a well-established screening instrument that has mostly been applied in non-teaching samples. In the current study, the psychometric properties of the questionnaire were analyzed using a large sample of German teachers (*N* = 3996). The data was collected yearly over an extended period of time (2012–2020). Results showed good to very good reliability, as well as high correspondence to burnout and life satisfaction scales. Principal axis factor analysis supported a two-factor structure: Factor 1 represents “depression/stress” and Factor 2 represents “loss of confidence”. However, the mental health of the investigated teachers was worse than that of a representative sample in Germany. Consequently, this study highlighted the fact that the teaching profession is vulnerable to mental strain and underlined the importance of promoting prevention programs that could help to sustain and foster the mental health of teachers. In this context, the GHQ-12 could be proposed as a good and economic tool to assess and analyze mental health in German teachers. The presented norm could help practitioners and teachers to compare individual scores within a larger peer group.

## 1. Introduction

Teachers play a key role in today’s society. They help students to learn by providing knowledge but also educate by conveying values and ethical standards. In this way, they support children, teenagers and young adults in their personal and professional growth as they become the future backbone of society. Therefore, the teaching profession is of paramount importance and each country should take care of the physical and mental health of their teachers. However, the reality is that teachers do not receive enough credit for their work [1,2] and findings around the world have shown that the state of their (mental) health is seriously concerning. Studies in Brazil and the UK have reported very high numbers of sick days among school teachers [3,4]. It has also been reported that one third of junior school teachers in the UK leave the profession within the first 5 years [5]. This corresponds to findings from a study in China, in which more than 40% of teachers stated that they would like to change their job if they could. The reasons that were given included a high level of distress, low salary, inadequate breaks and holidays, heavy workload, and student behavior” [6]. Similarly, two other reviews found that time pressure, high workload and additional administrative work were prominent reasons for teachers’ distress [7,8]. In addition, negative experiences in relationships with students, parents and colleagues were identified as the main factors for teachers’ mental health issues in several studies [9,10,11,12,13,14,15].

Studies from Germany have shown that teachers have higher rates of mental and psychosomatic disorders than other professions [16,17,18]. Seibt and Kreutzfeld [19] found that almost 50% of more than 11,000 German teachers in upper-level secondary schools reported burnout symptoms. A national survey in Germany also assessed that only 30% of teachers stay in their job until retirement and that 13% of those teachers who take early retirement leave their work because of psychosomatic disorders [20].

The OECD summarized this situation by stating that “teaching is a demanding profession with high levels of occupational stress” [21]. Sadly, the situation seems to be getting worse [22]. It is also evident that the COVID-19 pandemic has led to additional stress for teachers worldwide (e.g., [23,24,25,26,27]). This seems particularly critical as there is evidence of a correlation between the teachers’ (mental) health, the quality of their teaching [28,29] and the social–emotional development of their students [30,31]. Therefore, the protection of teachers’ health should be a main priority, not only to ensure the availability of enough teaching staff but also to provide a good education for healthy students. Adequate approaches to improve structural conditions, as well as to foster personal resources and strengthen individual resilience, should be identified and implemented. First, the mental health of teachers needs to be assessed regularly using an economically viable and reliable instrument and second, adequate training programs that significantly improve mental health should be implemented.

Since 2012, our team at the university hospital of Freiburg in Germany has been providing the “Manual-Based Psychological Group Program” (Lehrer-Coaching nach dem Freiburger Modell [32]), which aims to maintain and enhance teachers’ mental health. It is currently offered to all state-employed teachers in the German state of Baden-Wuerttemberg and up to now, more than 6000 teachers have participated. The program focuses on social support, as well as relationships, and uses the method that was proposed by Balint [33] to reflect on challenging and distress-causing cases. In addition, it is enriched with theoretical input on the neuroscientific aspects of health, identification and relationship building, as well as a method for systematic relaxation. The positive outcomes of this program are clearly evidenced [34,35]. For over 10 years, the General Health Questionnaire (GHQ-12) [36] has been used for evaluation purposes. The GHQ-12 is a well-established 12-item screening instrument that is used to assess mental health. It has been translated into various languages and successfully applied in numerous settings (e.g., [37,38,39,40,41,42,43]). However, there is still an open question as to whether the GHQ-12 could be proposed as an adequate instrument to monitor the state and development of teachers’ mental health. There is also an ongoing discussion about the psychometric properties of this measurement. Its reliability, sensitivity and specificity are considered to be good to very good [40]. The factor structure, in particular, has been discussed and re-evaluated in several samples from different countries. Initially, the instrument was created as a short version of the GHQ-60 and was proposed as being unidimensional [36]. Recently, some studies claimed to have confirmed the unidimensionality of the GHQ-12 [43,44,45,46,47], while other studies detected two [39,48], three [37,42,49,50,51] or even four factors [52]. In line with the factor names of the other GHQ versions and after reviewing the previous studies, Romppel et al. [53] suggested that in the two-factor structure, the factors could be defined as (i) “anxiety/depression” and (ii) “social dysfunction” and in the three-factor structure, the third component could be titled (iii) “loss of confidence”. Interestingly, the last factor seems to be unique to the GHQ-12 as it is not found in the factor structure of the GHQ-28 (e.g., [54,55]).

The differences in the factor structure became even more vivid in a study by Gelaye et al. [56], which compared results from different countries. The authors found a two-factor structure in the Chilean, Thai and Ethiopian samples and a three-factor structure in the Peruvian sample. Notably, even among the studies that identified the same number of factors, the order of the extracted components and the questions that belonged to each component were different between the samples. Furthermore, the sample sizes, as well as the sample properties, have been very heterogeneous between studies. These results suggest that the factor structure of the GHQ-12 questionnaire is influenced by the culture and structure of the investigated population (see [55] for a discussion on the GHQ-28). Therefore, it is important to continue investigating the psychometric properties of the GHQ-12 in different populations and larger samples.

To our knowledge, until now, only a handful of studies have investigated the GHQ-12 properties in a German sample (e.g., [43,53,57]). Additionally, none of these studies have examined the statistical properties of the GHQ in a sample of German teachers. The present study aimed to fill this research gap by using and analyzing the GHQ-12 in a large sample of German teachers. The study targeted the question of whether the GHQ-12 questionnaire could be a good screening instrument to assess the mental health of German teachers. Specifically, the reliability, factor structure and criterion validity of the German version were investigated. Regarding the criterion validity, we chose two concepts that theoretically have a high overlap with general mental health and are of high relevance to teachers: burnout and life satisfaction. As part of the burnout syndrome, emotional exhaustion is often the first sign of diminished mental health. The subscales are assumed to be highly associated with depression (here: emotional exhaustion) and a loss of confidence (here: cynicism, professional efficacy). In contrast, life satisfaction can be described as the opposite of depression and/or loss of confidence. Lastly, we aimed to contextualize our data by creating norm values for the large teacher population. Thus, the present study not only contributes to the ongoing scientific discussion about the psychometric properties of the inventory but also provides insights into teachers’ mental health in Germany.

## 2. Method

### 2.1. Sample and Design

The cross-sectional sample consisted of 3996 German teachers and was obtained within the context of a larger intervention/coaching study of school teachers in public schools in southern Germany (for more information about the coaching program, see [34,35]). Only participants who indicated that they were participating for the first time were included in the current study sample (filter question). Most of the participants were female (81.6%), older than 55 years of age (25.9%), had no leadership role (88.2%) and worked as a teacher full time (49.3%). The sample comprised teachers from a variety of school types (Table 1). Participation in the questionnaire was voluntary and anonymous. No incentive was offered for participation other than the opportunity to participate in a free group coaching session, according to Bauer [32]. The applied questionnaire was approved by the ethics committee at Freiburg University.

### 2.2. Procedure

At the beginning of each school year, before participating in the group coaching sessions, all participants were asked to fill out a questionnaire regarding demographics, health and other work-related variables. The data were collected over an extended period of time from 2012 to 2020 (most of the data were collected prior to the outbreak of the COVID-19 pandemic (*N* = 3777 vs. *N* = 219) and in our sample, there were no differences between the GHQ-12 scores that were collected before and during the COVID-19 pandemic). Some variables were only collected in certain years (e.g., MBI-D, AVEM, etc., as explained below). The data was obtained anonymously and participation was voluntary. By completing the questionnaire, informed consent was given by the participants to use the data for research purposes. The response rate varied between 49% (school year 2020/2021) and 94% (school year 2012/2013) for all school years (2012/2013 to 2020/2021).

### 2.3. Instruments

The German version of the General Health Questionnaire (GHQ-12 [36]; German version [58]; see [57] for more information) was applied. The current mental health status of the participants was assessed by the 12 items on a 4-point Likert scale (scores of 0, 1, 2 or 3). The total score ranged from 0 to 36, with lower scores representing better mental health and higher scores indicating higher levels of mental distress. 

The Maslach Burnout Inventory [59] was used in its German version [60] to assess the criterion validity. The German version was adapted to fit the teaching environment (MBI-D). Feelings of burnout were measured using three subscales: emotional exhaustion (nine items), cynicism/depersonalization (five items) and professional efficacy (eight items). All items were evaluated on a 6-point frequency rating scale (from 0 = never to 5 = very often). High values for exhaustion and depersonalization items and low values for professional efficacy items were considered as indicators of burnout. The MBI-D was only assessed during the first year of the intervention study (independent subsample; *n* = 793). The internal consistency was α = 0.87 for emotional exhaustion, α = 0.75 for cynicism/depersonalization and α = 0.80 for professional efficacy.

The subscale general life satisfaction (LS) from the work-related behavior and experience pattern scale (“Arbeitsbezogene Verhaltens- und Erlebensmuster”; AVEM-44, [61]) was also applied to measure the criterion validity. The four items were answered on a 5-point Likert scale (from 1 = strongly agree to 5 = strongly disagree). The scale was only assessed in later (school) years (independent subsample; *n* = 3203). The internal consistency was α = 0.86 for the current sample.

Demographic and teaching-relevant variables were obtained as well (Table 1 and Table 2). The demographic variables and GHQ scores of the two individual subsamples are presented in Table S1 in the Supplementary Materials.

### 2.4. Analyses

All analyses were conducted using IBM SPSS Statistics 28 (IBM Corp., Armonk, NY, USA). Due to missing values for different items of the GHQ-12 (Table 3), the analyses concerning factor structure were conducted on a sample of 3909 participants. As expected, there were some errors or common variance between items. Therefore, for the Principal Axis Factor analysis (PAF), an oblimin procedure was used to investigate the factor structure of the GHQ-12. The promax rotation with Kaiser normalization was chosen due to the large sample size. As the current sample size was over 200, a scree plot helped to determine the number of factors. In addition, structural equation modeling (SEM) using AMOS 28 (IBM Corp.; Armonk, NY, USA) was utilized to assess the structural fit of the different factor solutions. The kurtosis for all items was below the value of seven and the skewness was below three (Table 3), which suggested adequate normality. Thus, the maximum likelihood algorithm was applied to assess the fit of the proposed models. Several fit indices were calculated to examine the model fit: the comparative fit index (CFI), the standardized root mean square residual (SRMR) and the root mean square error of approximation (RMSEA). CFI values of 0.90 or greater and SRMR and RMSEA values of 0.05 or less indicated a good fit of the models [62,63]. Two measures of comparative fit were reported as well: the Akaike information criterion (AIC) and the Bayesian information criterion (BIC). For the AIC and BIC, lower values indicated a better fit of the model.

Before conducting the regression analyses, the categorical variables of tenure and teaching load were dummy coded. Moreover, multicollinearity was also tested. No multivariate outliners were identified using Mahalanobis distance, average leverage and Cook’s distance. To assess the amount of explained variance of the three subscales of burnout (MBI) and life satisfaction (AVEM), two stepwise multiple linear regression analyses in two independent subsamples were conducted with the GHQ score as dependent variable (the GHQ-12 score with the original 12 items was used; however, regression analyses that used GHQ-11 yielded the same results). The independent variables were entered in three blocks. First, the regression models were adjusted for age and gender in Step 1. In Model 1, teaching-related variables were added (Step 2: tenure, teaching load and leadership role). In Model 2, burnout and life satisfaction were added, respectively (Step 3: emotional exhaustion, depersonalization and professional efficacy vs. general life satisfaction). The unstandardized estimates (B), the standard error for B (*SE*), the lower and upper 95% confidence limits for B, the standardized estimates (*β*) and the level of significance for B were all reported. Adjusted *R^2^*, Δ*R^2^* and Δ*F* values were also presented to indicate the model fit and explained variance.

## 3. Results

### 3.1. Psychometric Properties and Reliability

When looking at the item statistics, the omission rate was very low and only four items had 10 or more missing values: “reasonably happy” (14 omissions), “lose sleep due to worry” (12 omissions), “loss of self-confidence” (11 omissions) and “face up to problems” (10 omissions). Items showed a large range of mean scores, with “feel worthless” (*M* = 0.68, *SD* = 0.80) having the lowest result and “constantly under strain” (*M* = 1.70, *SD* = 0.75) having the highest agreement. The standard division was relatively constant between the items (see Table 3).

The reliability analyses showed a good to excellent internal consistency, with a Cronbach’s α = 0.88. By looking at the results more closely, it was revealed that removing Item 5 (“face up to problems”) from the GHQ scale would lead to an increase in reliability to a Cronbach’s α = 0.90. Using MacDonald’s omega to calculate reliability yielded the same results of Ω = 0.88 and Ω = 0.90, respectively. Thereafter, the internal consistency could not be improved further by removing any of the other items.

### 3.2. Factor Structure

A Principal Axis Factor analysis (PAF) was conducted on the 12 GHQ-12 items using oblige rotation (promax). The Kaiser–Meyer–Olkin measure confirmed the adequacy of the sampling for this analysis, with KMO = 0.92 (which was excellent, according to Field [64]). However, five items (Items 1, 3, 4, 5 and 6) showed individual KMO values that were under the acceptable limit of 0.5. In particular, Item 5 had a value of KMO = 0.00. The results from Bartlett’s test of sphericity indicated that the correlations between the items were satisfactorily large for a factor analysis (*χ*^2^ (66) = 19689.83, *p* < 0.001). An initial analysis was run to obtain the eigenvalues for each component.

Three factors had eigenvalues over Kaiser’s criterion of 1 and explained 63.45% of the variance: Factor 1 (45.65%), Factor 2 (9.15%) and Factor 3 (8.66%). The scree plot showed indications that would justify retaining either two or four factors. Given the large sample size and the junction of the scree plot, two factors were retained in the final analysis. The pattern matrix of the factor loadings after rotation showed that Item 6 loaded on both factors above 0.3 and Item 5 did not load on either of the two factors (Table 4; see Table S2 in the Supplementary Materials for the structure matrix). Thus, the factors were not distinct and could not be interpreted easily.

Reliability analysis, as well as the factor analyses, suggested that Item 5 did not relate sufficiently to the rest of the items. Therefore, an additional factor analysis was run without this item, i.e., it was run on only 11 of the GHQ-12 items. Results were similar to those of the initial analyses: KMO = 0.93; four items (Items 1, 3, 4 and 6) showed individual KMO values of under 0.5; the results from the Bartlett’s test of sphericity were significant (*χ^2^* (55) = 19617.00, *p* < 0.001); and the scree plot and Kaiser’s criterion (>1) suggested two distinct factors. After the extraction, the two factors explained a variance of 51.56% (45.62% and 5.94% each). The pattern matrix of the factor loadings after rotation showed that each item loaded primarily on one factor (Table 5; see Table S3 in the Supplementary Materials for the structure matrix). The items that clustered on the same factors suggested that Factor 1 represents “depression/stress” and Factor 2 represents a “loss of confidence”. Both factors showed a good to excellent internal consistency.

In addition, structural equation modeling (SEM) was used to assess the structural fit of the three factor models: Model 1 used all items on one factor(unidimensional), Model 2 was unidimensional (without Item 5) and Model 3 used two correlated factors that were proposed by the exploratory factor analyses. Table 6 presents the fit statistics of these models. The unidimensional models (Models 1 and 2) did not present very good data fits. In addition, there was again no relationship between the latent variable of the GHQ-12 and Item 5 (*β* = −0.01) and the model fit indices did not improve by excluding Item 5 (CFI = 0.88, SRMR = 0.054 and RMSEA = 0.104 vs. CFI = 0.88, SRMR = 0.056 and RMSEA = 0.113). The model resembling the factor structure that was proposed by the factor analysis (see above, Model 3) showed a good to acceptable fit to the data (CFI = 0.94, SRMR = 0.04 and RMSEA = 0.08). In addition, AIC and BIC showed the lowest scores. The SEM also proposed the two-factor solution as the best fit.

### 3.3. The Role of Burnout and Life Satisfaction in General Psychological Health (GHQ-12)

To investigate the relationship between mental health measured by the GHQ-12 and the other measures of psychological health (burnout and life satisfaction), stepwise multiple linear regression analyses with the GHQ-12 as a dependent variable were conducted in two independent subsamples (Table 7 and Table 8).

In the first subsample (*n* = 793), the control variables (Step 1: gender and age) and the teaching-related variables (Step 2: Model 1) only explained a very small proportion of the variance in general psychological health (1%). By adding the three burnout symptoms (emotional exhaustion, depersonalization and professional efficacy) in Model 2, the total model became significant (*F*(12, 616) = 27.73, *p* < 0.001) and the explained variance increased significantly (*R*^2^ = 0.34, adjusted Δ*R*^2^ = 0.33). However, only leadership role (*β* = 0.08, *p* < 0.05) and emotional exhaustion (*β* = 0.56, *p* < 0.001) were significant predictors for general psychological health (Table 7). 

When conducting the same multiple linear regression model on the two factors “depression/stress” and “loss of self-confidence”, which were identified by the principal axis factor analysis, similar results were found with regard to the control and teaching-related variables (Model 1; Tables S4 and S5 in the Supplementary Materials). However, professional efficacy became a significant predictor in addition to emotional exhaustion for both factors. Interestingly, professional efficacy had a positive relationship with Factor 1 “depression/stress” and a negative relationship with Factor 2 “loss of self-confidence” (*β* = 0.08, *p* = 0.05 and *β* = −0.12, *p* < 0.01, respectively).

For the multiple linear regression analysis on the larger subsample (*n* = 3203), the results for the control variables and teaching-related variables (Model 1) again only explained a very small proportion of the variance in general psychological health (1%). However, in Model 2, life satisfaction yielded a significant total model (*F*(10, 2933) = 95.44, *p* < 0.001), as well as an increase in explained variance (*R*^2^ = 0.24, Δ*R*^2^ = 0.23). Again, only leadership role (*β* = 0.04, *p* < 0.05) and life satisfaction (*β* = −0.49, *p* < 0.001) were significant predictors for general psychological health (Table 8).

When conducting the same multiple linear regression analyses on the two factors that were identified by the Principal Axis Factor analysis, the results yielded a similar outcome as those for the global GHQ-12 factor (Tables S6 and S7 in the Supplementary Materials). However, with regard to Factor 1 (“depression/stress”), the teaching load dummy variables became significant predictors as well (Table S6 in the Supplementary Materials).

### 3.4. Stanine Standardization of the General Health Questionnaire

As the current study involved a very large sample of teachers in Germany (*N* = 3909 participants who answered *all* 12 questions), we transformed the GHQ-12 sum score (range = 0–36) into stanine scores using a percentage rating (Table 9). The mean score of the GHQ-12 sum score was 14.48 (SD = 5.93) and the mode was 12. Participants with GHQ sum scores of over 15 (> stanine 5) showed a worse mental health state than half of the norm sample.

## 4. Discussion

The aim of this current study was to (1) investigate the psychometric properties of the GHQ-12 in a larger sample of German teachers (*N* = 3996) and (2) assess the mental health of teachers in Germany. To achieve both of these aims, we used a cross-sectional dataset of German teachers who completed the GHQ-12 together with demographic and other variables in preparation of a prevention program. In this section, we first discuss the conceptual and methodological implications of our findings for the GHQ-12 and teachers’ health. We then state the possible limitations of the study and before finally discussing the applied implications for the mental health of teachers.

### 4.1. Psychometric Properties of the GHQ-12 as a Measurement for the Mental Health of Teachers

Firstly, the low omission rate suggested that the items of the German GHQ-12 had a high acceptance rate, i.e., they were easy to read, understand and answer. The reliability analyses for the GHQ-12 as a global factor also suggested that the items had good to very good internal consistency, particularly when Item 5 (“Face up to problems”) was removed. This was in line with previous findings, which have attested that the GHQ-12 has good reliability and acceptance (e.g., [40]; for the German version, see [53]).

Although the internal consistency was good to very good for the whole GHQ-12 questionnaire (12 items), our Principal Axis Factor analysis and SEM revealed that two factors were the best fit for the current data. It is important to note that in both analyses, Item 5 did not show a good relationship with the rest of the items. After removing Item 5 from the factor analyses, the items of the two extracted factors loaded > 0.5 on their respective factors with cross-loadings of < 0.3 (with one exception each). However, after removing Item 5 from the SEM model, only a small difference could be observed regarding the fit indices of the two models. Notably, the items of each of the two factors were related in content and could be interpreted according to previous factor solutions (e.g., [53]). The items “The items: “Lost sleep over worry”, “Constantly under strain”, “Could concentrate”, “Could not overcome difficulties”, “Reasonably happy”, “Enjoying day-to-day activities”, and “Unhappy and depressed” can be grouped under Factor 1 (“depression/stress”). These items matched the key components of depression (ICD-10, F 32): low mood, reduced capacity for enjoyment and interest and marked tiredness (even after minimum effort). Then, the items “Play useful part in things”, “Capable of making decisions”, “Lost self-confidence”, and “Felt worthless” can be clustered under Factor 2 (“loss of confidence”). Both factors showed a high internal consistency (α = 0.87 and α = 0.79, respectively). The misfit of Item 5 in the current dataset underlined the fact that the sample structure and specificity could influence the GHQ structure. Although Item 5 showed an acceptable mean and standard division score, it did not relate closely to the rest of the items and did not provide any additional explanation for the mental health of teachers. It could be hypothesized that the content of that item (“Face up to problems”) was already assessed by one or more of the other items (e.g., “could not overcome difficulties”), so the item did not generate any beneficial information. Moreover, it could also demonstrate a culture difference as “Face up to problems” is harder and less common in German than in English. Future studies should investigate whether particular aspects or questions within the GHQ may not apply to certain participants and/or contexts.

Both factors included positively and negatively worded items. As already discussed by previous authors, the two factors only distinguished between negatively and positively worded items and thus, were only a statistical and artificial solution and not a “real” two-factor solution [43,45]. It has been argued that the different response categories for the negatively and positively worded items were responsible for two-factor solutions. However, this was not the case in the current study, as can be seen from the composition of the factors and the results regarding the criterion validity. The stepwise regression analyses for the role of burnout in mental health (as measured by the GHQ-12) showed different results for professional efficacy in the global unidimensional factors, Factor 1 and Factor 2. First, the relationship between professional efficacy and mental health was non-existent when using the unidimensional GHQ-12 solution as a dependent variable. Conversely, when investigating both Factor 1 and Factor 2 as dependent variables, the regression coefficient of professional efficacy became substantial in each regression model. However, the direction of the relationship was positive for Factor 1 but negative for Factor 2, which suggested that professional efficacy played a different role in Factor 1 (“depression/stress”) and Factor 2 (“loss of confidence”). In addition, leadership role seemed to only influence Factor 1 (“depression/stress”) and not Factor 2. In sum, the results suggested that the two factors measured two rather distinct aspects of mental health.

In terms of criterion validity, the current study showed that mental health, as measured by the GHQ-12, was significantly related to burnout symptoms and life satisfaction. Emotional exhaustion was revealed as a strong predictor for the GHQ-12, especially for Factor 1. This was not surprising and in line with the theoretical assumptions, as well as previous research, which has shown that emotional exhaustion is the core facet of burnout that influences key aspects of mental health, as measured by the GHQ-12 (e.g., worry, depression, concentration and making decisions [65]). In line with the assumption of the criterion validity, life satisfaction also played a medium to large role in the GHQ-12. The negative correlation was in agreement with the concept of decreased mental health (high GHQ-12 scores) leading to decreased life satisfaction (low scores on the life satisfaction scale). The high influence of both measures on the GHQ-12 (as well as Factors 1 and 2) proved the criterion validity of the German version of the GHQ-12 as it intercorrelated strongly with the related concepts.

### 4.2. Mental Health of Teachers in the Current Sample

Compared to the study by Romppel et al. [53], the mental strain that was found in the current teaching sample was considerably higher. The GHQ-12 item scores of the current sample differed substantially from those of the representative German sample, which was investigated in 2012 (pre-COVID-19; *N* = 2041) [43,53]. In the present study, most of the mean scores were noticeably higher than the mean scores of the representative sample. This was in line with the research that has attested that teachers suffer from diminished mental health, as well as higher risks of psychological diseases and burnout (e.g., [18,19]). In fact, the 252 early childhood teachers in Korea who participated in the study by Lee and Kim [51] showed much higher item scores than the sample in the current study. In contrast, an earlier questionnaire study in southern Germany found lower GHQ scores (*M* = 12.25, *SD* = 5.08), thus a better psychological health [10]. However, the authors acknowledged that their data might “underestimate the professional strain of teachers” (p. 447) as other research has shown that teachers who decide against participating in questionnaire studies may be more distressed. As the current dataset was not purely a questionnaire study but a byproduct of a prevention study, the presented GHQ scores could include more participants who would not normally participate in questionnaire studies due to workload or exhaustion.

A recent study of German student teachers (teachers in their probation and training years) found a similar low mean score for psychological health (GHQ-12 scores for females: *M* = 12.5, *SD* = 6.59; GHQ-12 scores for males: *M* = 13.2, *SD* = 5.22) [66]. Similar to this result, the GHQ score increased with age in our sample as well. The younger teachers (<35 years of age) showed the lowest GHQ-12 score. This was in correspondence with the age-related hypothesis that psychological health decreases with age (e.g., higher burnout risk [67]). In addition, it has been argued that an increase in physical challenges and changes might be a main cause mental health risks in older age and that working conditions (e.g., demands, teaching load) that deplete resources may also contribute to that. Thus, tenure was found to be a significant predictor of psychological health in the current sample. Interestingly, a study by Darius et al. (2021) also indicated that the GHQ-12 score increased with study/teaching experience, with the highest score being observed during the exam period (*M* = 15.3, *SD* = 8.34). This score was even higher than those that we found in our study.

To summarize, the current study offered a unique insight into the psychological health of a large sample of German teachers, which could have incorporated participants who would not normally choose to be included in such research. The results suggested that mental health was in fact worse in German teachers than other populations. Age, tenure and gender could play an important role in this. From a psychometric perspective, this underlined the fact that the origin and structure of the sample population needs to be considered when investigating the structure and/or properties of the GHQ-12. Furthermore, specific norms are needed to help participants to classify their results in relation to their peer group.

## 5. Limitations

When generalizing and interpreting the results, some limitations needed to be considered. First, it could be argued that the results were limited to the current sample as participants who enroll in prevention studies may come from specific backgrounds. However, with some exceptions, the sample was fairly representative with regard to the demographics and teaching-related variables of teachers in southern Germany and/or Germany [19,68]. Moreover, although the study only included around 4.2% of the total number of teachers, the sample size was sufficiently large to run all of the conducted analyses robustly.

Second, the cross-sectional design did not allow for casual interpretation of the conducted regression analyses. As we merely wanted to state the current health status of the participating teachers, the cross-sectional design should be sufficient. However, future studies that are interested in looking into the differences between school types, for example, should favor longitudinal designs.

Third, mental health was measured via self-reporting, which could be considered as a limitation. However, the aim of the study was to investigate the use of a screening instrument for psychological health and teachers are obviously experts in their own mental well-being. Future studies could use more objective criterion measures to measure psychological health, such as archival data (e.g., sickness leave, performance measures, etc.), third-party reports (e.g., ratings from students, colleagues or supervisors) or biological stress markers (e.g., hair cortisol, etc.).

Fourth, there has been recent criticism of the use of the MBI as a valid scale to measure burnout (e.g., [69]). In the current study, the MBI version that is specific to educators was applied in the first year of the project (2012). During that time, the MBI was still considered the gold standard for measuring burnout. Furthermore, to our knowledge, this is the only commonly used and evaluated burnout measure that has been adopted specifically for educational setting. For these reasons, the MBI was used in the current study. Nevertheless, future studies should use other burnout measures (e.g., OLBI [70]) to assess and confirm the criterion validity of the GHQ-12.

Lastly, it could be argued that the analyses and norm should have been calculated separately for each school type. We acknowledge that students in each school type are different. However, the organization and education of teachers is very structured in Germany. Therefore, in line with the findings of previous studies (e.g., [18]), we hypothesized that there were no significant differences between the different school types in regard to teachers’ general mental health to justify separate analyses. Nevertheless, future studies should focus on the mental health of teachers in different school types and/or states/countries.

## 6. Conclusions & Practical Implications

The German version of the GHQ-12 proved to be a good (valid) and reliable tool to measure mental health or strain of teachers, as well as other subgroups. Through the screening character, it is a time-efficient way to assess the mental health of individual teachers, subgroups of teachers or the whole body of teachers. Thus, it can be easily included in yearly, or even quarterly, assessments to regularly monitor the health status of the teaching body and identify stress peaks. With the norm presented in this paper, (German) teachers now have the opportunity to benchmark themselves in relation to others in their peer group, which could help to interpret individual results more easily. It can be suggested, that groups and individual teachers that are above the average (>stanine 5, GHQ-Score 15) show a diminished or critical mental health. These teachers with above average scores need to be informed and health promotion programs should to be suggested. In addition to the presented norm, a cut-off procedure is often also suggested for the GHQ-12. However, Goldberg et al. [71] pointed out early on that the threshold also varies between countries and samples. Here, the norm offered might be a more sensitive and practical way to rank individual teachers and their superiors. As the GHQ-12 is sensitive to different cultures, teachers and authorities from other countries should apply the presented norm with caution. Future studies should attempt to calculate norms for educators in different countries and under post-COVID-19 conditions.

However, the norm does not take away from the fact that teachers seem to be under more mental strain than the average population in Germany [53] and that prevention is necessary to sustain and foster the mental health of teachers. Society (e.g., the responsible political institutions) needs to find and establish ways to promote teachers’ mental health and decrease mental strain. This is particularly true under the increased workload and changes that have occurred during the COVID-19 pandemic. Recent studies have shown that the pandemic may lead to an even greater risk in mental health [72,73]. The GHQ-12 is an efficient way to provide an overview of mental strain, bring transparency and help self-reflection. Groups or individuals who are at risk could be identified and supported using the GHQ-12. In addition, for professional occupational therapists, it could be beneficial to distinguish between the two factors “depression/stress” and “loss of confidence” as this distinction may assist in finding the key points for sustaining or fostering the mental health of the client, as well as assigning the right preventative measures. In sum, the GHQ-12 can be proposed as a very useful and efficient instrument for the exploration of teachers’ mental health in Germanyand with its specific norm other countries as well.

## Figures and Tables

**Table 1:** The demographic variables and GHQ scores of the study sample.

	Study Sample		GHQ Score		z-/H-Value
*N*	3996		14.48 (5.93) ^1^		
*Demographics*					
Gender					−2.47 **
female	3189 (81.6%)		14.51 (5.88)		
male	716 (18.3%)		14.04 (6.00)		
Age (years)					39.50 ***
<35	332	(8.3%)	13.57	(5.78)	
35–39	420	(10.6%)	13.80	(5.98)	
40–44	689	(17.3%)	14.22	(5.83)	
45–49	734	(18.4%)	14.11	(5.81)	
50–54	775	(19.5%)	14.83	(5.92)	
>55	1030	(25.9%)	15.23	(6.01)	
Relationship status					−5.21 ***
no	783	(19.9%)	15.50	(6.11)	
yes	3148	(80.1%)	14.19	(5.84)	
Tenure (years)					31.97 ***
<10	865	(21.7%)	13.95	(6.08)	
10–14	1064	(26.7%)	14.40	(5.77)	
15–19	779	(19.5%)	14.01	(5.71)	
>20	1279	(32.1%)	15.18	(6.03)	
Teaching load					4.67
100%	1960	(49.3%)	14.29	(6.00)	
>75%	839	(21.1%)	14.69	(5.88)	
50–75%	971	(24.4%)	14.62	(5.76)	
<50%	206	(5.2%)	14.57	(6.28)	
Leadership responsibility					−0.50
No	3330	(88.2%)	14.43	(5.92)	
Yes	446	(11.8%)	14.56	(6.02)	
School type					19.55 **
Basic elementary school (1st–4th year)	943	(23.6%)	14.69	(5.88)	
Secondary school (5th–9th year)	331	(8.3%)	14.87	(6.46)	
Secondary school (5th–10th year)	475	(11.9%)	14.44	(5.85)	
High school (5th–13th year)	905	(22.7%)	13.92	(5.85)	
Community school	117	(2.9%)	15.63	(6.37)	
Vocational school	702	(17.6%)	14.36	(5.90)	
special schools for mentally or physically handicapped pupils	472	(11.8%)	14.69	(5.64)	
Others	49	(1.2%)	15.54	(6.41)	

*Note*: *N* = 3776–3994; ** *p* < 0.01; *** *p* < 0.001; ^1^ due to missing values, the GHQ-12 sum scores were only available for 3909 participants.

**Table 2:** The correlations between the demographics and the health variables.

	1.	2.	3.	4.	5.	6.	7.	8.	9.	10.	11.
*Demographics*											
1. Age	1										
2. Gender	0.058 **	1									
3. Relationship Status	−0.062 **	0.077 **	1								
4. Tenure	0.742 **	0.036 *	−0.053 **	1							
5. Teaching Load	−0.122 **	−0.294 **	0.126 **	−0.165 **	1						
6. Leadership Role	0.134 **	0.041 *	0.037 *	0.194 **	−0.257 **	1					
*Health*											
7. GHQ (Sum)	0.092 **	−0.031	−0.088 **	0.070 **	0.024	0.007	1				
8. Emotional Exhaustion	0.091 *	−0.032	−0.088 *	0.086 *	0.041	−0.076 *	0.586 **	1			
9. Depersonalization	−0.053	0.199 **	−0.023	−0.034	−0.048	−0.036	0.293 **	0.479 **	1		
10. Professional Efficacy	−0.004	−0.086 *	0.043	0.061	−0.169 **	0.221 **	−0.251 **	−0.437 **	−0.493 **	1	
11. Life Satisfaction	−0.118 **	−0.011	0.248 **	−0.061 **	−0.005	0.089 **	−0.491 **	-	-	-	1

*Note:* * *p* < 0.05; ** *p* < 0.01; *N_(MBI)_* = 715–783; *N* = 3038–3971.

**Table 3:** The statistics for the GHQ-12 items.

		*N*	*M*	*SD*	Skewness/*SE*		Kurtosis/*SE*	
1.	Lost sleep over worry (N)	3984	1.33	(0.88)	−0.003	0.039	−0.795	0.078
2.	Constantly under strain (N)	3993	1.70	(0.75)	0.024	0.039	−0.478	0.077
3.	Could concentrate (P)	3987	1.38	(0.61)	0.730	0.039	0.242	0.078
4.	Play useful part in things (P)	3995	1.05	(0.62)	0.563	0.039	1.36	0.077
5.	Face up to problems (P)	3986	0.86	(0.72)	0.526	0.039	0.054	0.078
6.	Difficulty in decision making (N)	3993	0.98	(0.74)	0.414	0.039	−0.076	0.077
7.	Could not overcome difficulties (N)	3991	1.20	(0.78)	0.229	0.039	−0.371	0.077
8.	Reasonably happy (P)	3982	1.44	(0.76)	0.215	0.039	−0.295	0.078
9.	Enjoying day-to-day activities (P)	3987	1.52	(0.73)	0.205	0.039	−0.315	0.078
10.	Unhappy and depressed (N)	3988	1.26	(0.87)	0.154	0.039	−0.709	0.078
11.	Lost self-confidence (N)	3985	1.08	(0.81)	0.319	0.039	−0.495	0.078
12.	Felt worthless (N)	3991	0.68	(0.80)	0.929	0.039	0.054	0.077

*Note*: N, negatively worded item; P, positively worded item; *M*, mean; *SD*, standard deviation; *SE*, standard error; scale range = 0–3. The answer scale for positive worded items was inverted. Lower mean scores represented better mental health and higher scores indicated higher levels of mental distress.

**Table 4:** The pattern matrix for the principal axis factor analysis on all of the GHQ-12 items.

	Factor 1	Factor 2
Item 2	**0.890**	−0.246
Item 9	**0.785**	−0.087
Item 8	**0.743**	0.045
Item 1	**0.613**	−0.033
Item 7	**0.610**	0.192
Item 10	**0.604**	0.257
Item 3	**0.577**	0.041
Item 12	−0.015	**0.863**
Item 11	0.187	**0.654**
Item 4	0.109	**0.512**
Item 6	**0.318**	**0.336**
Item 5	0.027	−0.035
Eigenvalues	5.02	0.65
% of Variance	41.82%	5.46%

*Note: N* = 3909; 12 iterations were needed to extract two factors. The extraction method was principal axis factor analysis with promax rotation and Kaiser normalization. The rotation took three iterations to converge. Eigenvalues above 0.3 (bold) indicate relatedness to the respective factor.

**Table 5:** The pattern matrix for the principal axis factor analysis on 11 of the GHQ-12 items.

	Pattern Matrix	
	Factor	
	1	2
Item 2	**0.867**	−0.186
Item 9	**0.739**	−0.015
Item 8	**0.682**	0.123
Item 1	**0.575**	0.104
Item 7	**0.537**	0.269
Item 3	**0.527**	0.104
Item 10	**0.522**	0.341
Item 12	−0.154	**0.955**
Item 11	0.070	**0.741**
Item 4	0.017	**0.581**
Item 6	0.238	**0.405**
Eigenvalues	5.02	0.66
% of Variance	45.62%	5.94%
α	0.87	0.79
MacDonald’s Omega	0.87	0.81

*Note: N* = 3919. The extraction method was principal axis factor analysis with promax rotation and Kaiser normalization. It took 12 iterations to extract two factors and the rotation took three iterations to converge. Eigenvalues above 0.3 (bold) indicate relatedness to the respective factor.

**Table 6:** The statistics for the fit of the tested models of the GHQ-12.

Model	X^2^	*df*	CFI	SRMR	RMSEA	AIC	BIC
Model 1 (unidimensional with all items)	2351.91 ***	54	0.883	0.054	0.104	2399.91	2550.42
Model 2 (unidimensional without Item 5)	2230.92 ***	44	0.888	0.056	0.113	2274.92	2412.88
Model 3 (two correlated factors)	1229.68 ***	43	0.939	0.044	0.084	1275.82	1419.91

*Note: N* = 3909; *** *p* < 0.001; *df*, degrees of freedom; CFI, comparative fit index; SRMR, standardized root mean square residual; RMSEA, root mean square error of approximation; AIC, Akaike information criterion; BIC, Bayesian information criterion.

**Table 7:** The role of burnout in psychological well-being (stepwise hierarchical regression with GHQ-12 as a dependent variable).

	Model 1 (Teaching-Related Variables)					Model 2 (Burnout)				
	B	*SE*	95% CI	*β*	*p*	B	*SE*	95% CI	*β*	*p*
*Step 1: Covariates*										
Gender (Female (ref.)/Male)	−1.21	0.63	(−2.45; 0.03)	−0.08	^+^	−1.00	0.53	(−2.04; 0.04)	−0.07	^+^
Age	0.28	0.26	(−0.24; 0.79)	0.06		0.11	0.21	(−0.31; 0.53)	0.02	
*Step 2: Teaching-Related Variables*										
Tenure										
*<10 Years (ref.)*										
*10–14 Years*	−1.36	1.17	(−3.65; 0.92)	−0.10		−0.45	0.95	(−2.32; 1.43)	−0.03	
*15–19 Years*	−1.35	1.26	(−3.83; 1.12)	−0.09		−0.85	1.03	(−2.87; 1.17)	−0.06	
>*20 Years*	−1.17	1.32	(−3.77; 1.43)	−0.10		−1.10	1.08	(−3.22; 1.03)	−0.09	
Teaching Load										
*100% (ref.)*										
>*75%*	0.62	0.68	(−0.72; 1.96)	0.04		0.36	0.56	(−0.74; 1.46)	0.02	
*50–75%*	0.24	0.65	(−1.03; 1.52)	0.02		0.13	0.53	(−0.92; 1.17)	0.01	
*<50%*	−0.61	2.01	(−4.57; 3.34)	−0.01		−0.16	1.64	(−3.39; 3.06)	−0.00	
Leadership Role (No (ref.)/Yes)	0.40	0.68	(−0.94; 1.74)	0.03		1.25	0.56	(0.15; 2.36)	0.08	*
*Step 3: Burnout*										
Emotional Exhaustion						4.04	0.29	(3.47; 4.60)	0.56	***
Depersonalization						0.25	0.32	(−0.37; 0.87)	0.03	
Professional Efficacy						−0.21	0.40	(−1.00; 0.57)	−0.02	
Adjusted *R*^2^	0.001					0.33				
Adjusted Δ*R*^2^	0.00					0.34				
Δ*F*	0.40					106.02	***			

*Note: N* = 629; adjusted *R*^2^ = 0.008 for Step 1; Durbin–Watson statistic = 0.75; ref., reference category; ^+^ *p* < 0.1; * *p* < 0.05; *** *p* < 0.001.

**Table 8:** The role of life satisfaction in psychological well-being (stepwise hierarchical regression with GHQ-12 as a dependent variable).

	Model 1 (Teaching-Related Variables)					Model 2 (Life Satisfaction)				
	B	*SE*	95% CI	*β*	*p*	B	*SE*	95% CI	*β*	*p*
*Step 1: Covariates*										
Gender (Female (ref.)/Male)	−0.11	0.30	(−0.69; 0.47)	−0.01		−0.19	0.26	(−0.70; 0.32)	−0.01	
Age	0.36	0.10	(0.16; 0.55)	0.10	***	0.06	0.09	(−0.12; 0.23)	0.02	
*Step 2: Teaching-Related Variables*										
Tenure										
*<10 Years (ref.)*										
*10–14 Years*	−0.24	0.33	(−0.89; 0.40)	−0.02		−0.05	0.29	(−0.61; 0.52)	0.00	
*15–19 Years*	−0.82	0.39	(−1.58; −0.06)	−0.06	*	−0.53	0.34	(−1.19; 0.14)	−0.04	
>*20 Years*	0.03	0.44	(−0.83; 0.90)	0.00		0.42	0.39	(−0.34; 1.17)	0.03	
Teaching Load										
*100% (ref.)*										
>*75%*	0.26	0.29	(−0.31; 0.84)	0.02		0.03	0.26	(−0.48; 0.53)	0.00	
*50–75%*	0.33	0.29	(−0.23; 0.90)	0.03		0.42	0.25	(−0.08; 0.91)	0.03	^+^
*<50%*	0.49	0.48	(−0.45; 1.43)	0.02		0.56	0.42	(−0.26; 1.38)	0.02	
Leadership Role (No (ref.)/Yes)	−0.20	0.37	(−0.92; 0.53)	−0.01		0.74	0.33	(0.10; 1.38)	0.04	**
*Step 3: Life Satisfaction*										
Life Satisfaction						−3.63	0.12	(−3.86; −3.39)	−0.49	***
Adjusted *R*^2^	0.01					0.24				
Adjusted Δ*R*^2^	0.001					0.23				
Δ*F*	1.58					907.66	***			

*Note: N* = 2944; adjusted *R*^2^ = 0.008 for Step 1; Durbin–Watson statistic = 0.46; ref., reference category; ^+^ *p* < 0.1; * *p* < 0.05; ** *p* < 0.01; *** *p* < 0.001.

**Table 9:** The stanine normalization of the GHQ-12 (*N* = 3909 German teachers).

GHQ Score (sum)	Stanine	Percentage (in Population)
**0–5**	1	120 (3.07%)
**6–7**	2	306 (7.83%)
**8–9**	3	421 (10.77%)
**10–11**	4	507 (12.97%)
**12–15**	5	996 (25.48%)
**16–18**	6	643 (16.45%)
**19–22**	7	522 (13.35%)
**23–26**	8	249 (6.37%)
**27–36**	9	145 (3.71%)

## Data Availability

The datasets that were generated for this study are available upon request from the corresponding author. The data are not publicly available due to privacy reasons/agreement with the funding agencies.

## Supplementary Materials

The following are available online at https://www.mdpi.com/article/10.3390/ijerph19159708/s1, Table S1: Demographics variables for the two subsample used in the construct validity analyses, Table S2: Structure Matrix for Principal Axis Factor analysis with all 12 GHQ items, Table S3: Structure Matrix for Principal Axis Factor analysis with all 11 GHQ items, Table S4: The role of burnout on GHQ-Factor 1 “depression/stress” (stepwise hierarchical regression), Table S5: The role of burnout on GHQ-Factor 2 “loss of confidence” (stepwise hierarchical regression), Table S6: The role of life satisfaction on GHQ-Factor 1 “depression/stress” (stepwise hierarchical regression), Table S7: The role of life satisfaction GHQ-Factor 2 “loss of confidence” (stepwise hierarchical regression).
