# Supplementary material for: How to Measure the Mental Health of Teachers? Psychometric Properties of the GHQ-12 in a Large Sample of German Teachers
Source: Int J Environ Res Public Health. 2022 Aug 6;19(15):9708. doi: 10.3390/ijerph19159708 (PMC9368010; doi:10.3390/ijerph19159708)
Supplement: Supplementary file 1 [file ijerph-19-09708-s001.zip › ijerph-1783962-supplementary.pdf]

APPENDIX/Supplementary Materials.

Table S1

Demographics variables for the two subsample used in the construct validity analyses.

	Subsample MBI		Subsample LSS	
n	793		3203	
Demographics	*n*	%	*n*	%
Gender				
Female	577	72.8	2612	81.5
Male	147	18.5	569	17.8
Age (years)				
< 35	5	0.6	327	10.2
35-39	40	5.0	380	11.9
40-44	131	16.5	558	17.4
45-49	116	14.6	618	19.3
50-54	192	24.2	583	18.2
>55	309	39.0	721	22.5
Relationship status				
no	175	22.1	608	19.0
yes	588	74.1	2560	79.9
Tenure (years)				
< 10	46	5.8	819	25.6
10-14	207	26.1	857	26.8
15-19	153	19.3	626	19.5
>20	383	48.3	896	28.0
Teaching load				
100%	439	55.4	1521	47.5
>75%	155	19.5	684	21.4
50-75%	179	22.6	792	24.7
<50%	13	1.6	193	6.0
Leadership responsibility				
No	128	74.8	2737	85.5
Yes	593	16.1	318	9.9
School type				
Basic elementary school (1st-4th year)	194	24.5	749	23.4
Secondary school (5th- 9th year)	94	11.9	237	7.4
Secondary school (5th- 10th year)	87	11.0	388	12.1
High school (5th-13th year)	159	20.1	746	23.3
Community school	-	-	117	3.7
Vocational school	154	19.4	548	17.1
special schools for mentally or physically handicapped pupils	91	11.5	381	11.9
Others	13	1.6	36	1.1

Note. MBI: Maslach Burnout Inventory, LSS: Life satisfaction scale.

Table S2

Structure Matrix for Principal Axis Factor analysis with all 12 GHQ items.

	Factor 1	Factor 2
Item 10	.777	.664
Item 8	.773	.546
Item 7	.739	.603
Item 9	.726	.443
Item 2	.724	.355
Item 3	.604	.430
Item 1	.591	.381
Item 12	.568	.853
Item 11	.629	.780
Item 4	.455	.586
Item 6	.545	.550
Item 5	.004	-.016

Note. N = 3909, extraction method: Principal Axis Factor analysis with promax rotation and Kaiser-normalization.[a] It took 12 iterations to extract 2 factors. Rotation took 3 iterations to converge.

Table S3

Structure Matrix for Principal Axis Factor analysis with all 11 GHQ items.

	Factor 1	Factor 2
Item 8	.769	.606
Item 10	.763	.710
Item 2	.735	.427
Item 7	.728	.650
Item 9	.728	.507
Item 3	.601	.478
Item 1	.591	.430
Item 12	.522	.846
Item 11	.595	.791
Item 4	.428	.592
Item 6	.525	.574

Note. N = 3919, extraction method: Principal Axis Factor analysis with promax rotation and Kaiser-normalization.[a] It took 12 iterations to extract 2 factors. Rotation took 3 iterations to converge.

Table S4

The role of burnout on GHQ-Factor 1 "depression/stress" (stepwise hierarchical regression)

	Model 1 (Teaching-related variables)					Model 2 (Burnout)				
	B	*SE*	95% CI	*β*	*p*	B	*SE*	95% CI	*β*	*p*
Step 1: Covariates										
Gender (female(ref.)/male)	-.18	.06	(-.29; -.06)	-.13 **		-.12	.05	(-.22; -.02)	-.09 *	
Age	.03	.02	(-.02; .08)	.07		.01	.02	(-.03; .05)	.03	
Step 2: Teaching-related variables										
Tenure										
< 10 years(ref)										
10-14 years	-.05	.11	(-.26; .16)	-.04		-.00	.09	(-.18; .18)	.00	
15-19 years	-.05	.11	(-.28; .18)	-.04		-.03	.10	(-.22; .17)	-.02	
> 20 years	-.04	.12	(.73; -.28)	-.04		-.05	.10	(-.25; .15)	-.05	
Teaching load										
100% (ref)										
>75%	.05	.06	(-.08; .17)	.03		.03	.05	(-.07; .14)	.02	
50-75%	-.02	.06	(-.13; .10)	-.01		-.00	.05	(-.10; .10)	-.00	
< 50%	-.13	.19	(-.50; .25)	-.03		-.08	.16	(-.22; .17)	-.02	
Leadership role (no(ref.)/yes)	.06	.07	(-.06; .19)	.04		.12	.06	(.02; .23)	.08 *	
Step 3: Burnout										
Emotional exhaustion						.41	.03	(.35; .46)	.58 ***	
Depersonalization						-.00	.03	(-.06; .06)	-.00	
Professional efficacy						.07	.04	(-.00; .15)	.08 +	
Adjusted R^2	.01					.31				
Adjusted $\triangle R^2$	.00					.30				
$\triangle F$	.38					92.81	***			

Note. $N = 647$. Adjusted $R^2 = .02$ for Step 1. Durbin-Watson-Statistics = .97. ref.= reference category. $^{+} p < .1$, * $p < .05$, ** $p < .01$, *** $p < .001$.

Table S5

The role of burnout on GHQ-Factor 2 "loss of confidence" (stepwise hierarchical regression)

	Model 1 (Teaching-related variables)					Model 2 (Burnout)				
	B	*SE*	95% CI	*β*	*p*	B	*SE*	95% CI	*β*	*p*
Step 1: Covariates										
Gender (female(ref.)/male)	-.04	.06	(-.16; .08)	-.03		-.05	.06	(-.16; .06)	-.03	
Age	.02	.03	(-.03; .07)	.05		.01	.02	(-.04; .05)	.01	
Step 2: Teaching-related variables										
Tenure										
< 10 years(ref)										
10-14 years	-.22	.12	(-.45; .00)	-.16	+	-.12	.10	(-.31; .08)	-.09	
15-19 years	-.25	.12	(-.49; -.01)	-.17	*	-.18	.11	(-.38; .04)	-.12	
> 20 years	-.24	.13	(-.50; .01)	-.20	+	-.20	.11	(-.42; .02)	-.17	+
Teaching load										
100% (ref)										
>75%	.07	.07	(-.07; .20)	.04		.02	.06	(-.10; .13)	.01	
50-75%	.07	.06	(-.06; .20)	.05		.04	.06	(-.07; .15)	.03	
< 50%	.05	.20	(-.35; .44)	.01		.08	.17	(-.26; .43)	.02	
Leadership role (no(ref.)/yes)	-.03	.07	(-.17; .10)	-.02		.06	.06	(-.06; .18)	.04	
Step 3: Burnout										
Emotional exhaustion						.31	.03	(25.; .37)	.43	***
Depersonalization						.04	.03	(-.03; .10)	.05	
Professional efficacy						-.12	.04	(-.20; -.04)	-.12	**
Adjusted R^2	.00					.26				
Adjusted $\triangle R^2$	.01					.26				
$\triangle F$	1.16					77.21	***			

Note. $N = 647$. Adjusted $R^2 = .00$ for Step 1. Durbin-Watson-Statistics = 1.20. ref.= reference category. * $p < .05$, ** $p < .01$, *** $p < .001$.

Table S6

The role of life satisfaction on GHQ-Factor 1 "depression/stress" (stepwise hierarchical regression)

	Model 1 (Teaching-related variables)					Model 2 (Life satisfaction)				
	B	*SE*	95% CI	*β*	*p*	B	*SE*	95% CI	*β*	*p*
Step 1: Covariates										
Gender (female(ref.)/male)	-.03	.03	(-.09; .03)	-.02		-.04	.03	(-.09; .01)	-.03	
Age	.03	.01	(.01; .05)	.09	***	.01	.01	(-.01; .02)	.02	
Step 2: Teaching-related variables										
Tenure										
< 10 years (ref)										
10-14 years	-.02	.03	(-.09; .04)	-.02		-.01	.03	(-.06; .05)	-.00	
15-19 years	-.07	.04	(-.15; .00)	-.05	+	-.05	.03	(-.11; .02)	-.03	
> 20 years	.02	.04	(-.07; .10)	.01		.05	.04	(-.03; .12)	.04	
Teaching load										
100% (ref)										
>75%	.02	.03	(-.03; .08)	.02		.00	.03	(-.05; .05)	.00	
50-75%	.02	.03	(-.04; .07)	.01		.02	.03	(-.03; .07)	.02	
< 50%	.01	.05	(-.08; .10)	.00		.02	.04	(-.07; .10)	.01	
Leadership role (no(ref.)/yes)	.03	.04	(-.04; .10)	.02		.11	.03	(.05; .18)	.06	***
Step 3: Life satisfaction (AVEM-LZ)										
Life satisfaction						-.32	.01	(-.34; -.29)	-.44	***
Adjusted R^2	.01					.20				
Adjusted $\triangle R^2$	.00					.19				
$\triangle F$	1.43					688.12	***			

Note. N = 3001. Adjusted R^2 = .01 for Step 1. Durbin-Watson-Statistics = .66. ref.= reference category. * $p < .05$, ** $p < .01$, *** $p < .001$.

Table S7

The role of life satisfaction GHQ-Factor 2 "loss of confidence" (stepwise hierarchical regression)

	Model 1 (Teaching-related variables)					Model 2 (Life satisfaction)				
	B	*SE*	95% CI	*β*	*p*	B	*SE*	95% CI	*β*	*p*
Step 1: Covariates										
Gender (female(ref.)/male)	.04	.03	(-.02; .09)	.02		.03	.03	(-.02; .08)	.02	
Age	.03	.01	(.01; .05)	.09	**	.00	.01	(-.01; .02)	.01	
Step 2: Teaching-related variables										
Tenure										
< 10 years (ref)										
10-14 years	-.01	.03	(-.08; .05)	-.01		.01	.03	(-.05; .06)	.01	
15-19 years	-.06	.04	(-.90; -.16)	-.04		-.03	.03	(-.09; .04)	-.02	
> 20 years	.00	.04	(-.08; .08)	.00		.03	.04	(-.04; .11)	.03	
Teaching load										
100% (ref)										
>75%	.05	.03	(-.01; .10)	.03		.02	.03	(-.03; .07)	.02	
50-75%	.06	.03	(.01; .12)	.05	*	.07	.03	(.02; .11)	.05	**
< 50%	.10	.05	(.01; .19)	.04	*	.11	.04	(.03; .19)	.04	*
Leadership role (no(ref.)/yes)	-.11	.04	(-.18; -.04)	-.06	**	-.03	.03	(-.09; .04)	-.01	
Step 3: Life satisfaction (AVEM-LZ)										
Life satisfaction						-.34	.01	(-.37; -.32)	-.47	***
Adjusted R^2	.01					.23				
Adjusted $\triangle R^2$	.01					.22				
$\triangle F$	3.97	***				846.47	***			

Note. $N = 3001$. Adjusted $R^2 = .005$ for Step 1. Durbin-Watson-Statistics = .94. ref.= reference category. * $p < .05$, ** $p < .01$, *** $p < .001$.
